# Dosimetric comparison of simultaneous integrated boost with whole-breast irradiation for early breast cancer

**DOI:** 10.1371/journal.pone.0173552

**Published:** 2017-03-08

**Authors:** Seok Hyun Son, Kyu Hye Choi, Shin-Wook Kim

**Affiliations:** Department of Radiation Oncology, Incheon St. Mary's Hospital, College of Medicine, The Catholic University of Korea, Seoul, Republic of Korea; Northwestern University Feinberg School of Medicine, UNITED STATES

## Abstract

**Purpose:**

The purpose of this study was to identify a more suitable boost plan for simultaneously integrated boost scheme in patients with breast cancer by comparing among 3 types of whole-breast irradiation plus tumor bed boost plans.

**Methods:**

Twenty patients who received radiotherapy following breast-conserving surgery for early breast cancer were enrolled in this study. We performed 1 type of electron plan (E1P plan) and 2 types of 3-dimensional conformal plans using a photon (P3P and P5P plans). The dosimetric parameters for the heart, total lung and the target volume between the 3 treatment types were compared.

**Results:**

For the tumor bed, the difference in the mean dose between the 3 plans was maximally 0.1 Gy. For normal breast parenchyma, the difference in the mean dose between the 3 plans was maximally 1.1 Gy. In the dose range over the prescribed dose of 51 Gy, V_55_ and V_60_ in the E1P plan were lower than those in the P3P and P5P plans, which indicated that the E1P plan was more suitable than the P3P and P5P plans. In case of the heart and total lung, the values of clinically important parameters were slightly higher in the E1P plan than in the P3P and P5P plans. However, these differences were less than 2%.

**Conclusion:**

We observed that a simple electron plan for tumor bed boost is preferable over multi-field photon plans in terms of the target volume coverage and normal tissue sparing.

## Introduction

Breast cancer is the most common cancer found in women, and the main cause of death among women worldwide [1]. Adjuvant radiotherapy (RT) has been found to enhance both the local control of cancer and the overall survival in early breast cancer [2, 3]. Furthermore, additional boosts to the tumor bed have also been shown to be beneficial in reducing local recurrence rates [4, 5]. Therefore, RT following breast-conserving surgery (BCS) is currently the standard treatment for patients with early breast cancer. Although till now, whole-breast irradiation (WBI) followed by a boost to the tumor bed was commonly used for breast cancer treatment, recently, simultaneous integrated boost (SIB), which is a WBI technique wherein a concurrent boost is delivered to the tumor bed, has been reported to be technically and dosimetrically feasible [6–8].

Conventional RT, 3-dimensional RT (3D-CRT), dynamic conformal arc therapy (DCAT), intensity-modulated RT (IMRT), volumetric-modulated RT (VMAT), and proton therapy are used for WBI. For boosts to the tumor bed, relatively simple electron therapy and brachytherapy may also be used. In addition, there have been recent reports regarding the effectiveness of the hybrid technique, which is a combination of different techniques for WBI and boosts [6, 8, 9].

In previous studies on boost planning, multi-fields 3D-CRT, IMRT, or VMAT have been reported to be superior to electron therapy with respect to the target volume coverage and normal tissue sparing [9–13]. However, because the required dose to boost was approximately 15%-25% of the total dose needed for adjuvant RT, the adequacy of planning should not be assessed using the boost planning alone. Both WBI and boost planning should be assessed together, especially in the case of the SIB technique.

In this study, we performed 1 type of electron plan and 2 types of 3D-CRT plans using a photon to identify a more suitable boost plan for SIB in patients with breast cancer by comparing these plans.

## Materials and methods

### Patients

Twenty patients who received RT following BCS for early breast cancer were enrolled for this study. According to the location of the tumor bed, the patients were classified into the following group: central, superior, inferior, lateral, or medial portion. For each location group, 4 patients were selected (2 patients with left breast cancer and 2 patients with right breast cancer). Simulation computed tomography (CT) data were collected for this planning comparative study after institutional review board approval (IRB of Incheon St. Mary's Hospital, College of Medicine, The Catholic University of Korea, reference number: OC16RISI0140). IRB approved that this study was exempted from obtaining of written informed consent because of the retrospective nature of this study.

### Simulation

The selected patients were in the supine position on 10°-15° angle breast-tilting board with both the arms elevated and immobilized using Vac-Lock devices. The extent of the breast parenchyma was marked using radio-opaque non-metallic wire for the target volume delineation via palpation. Simulation CT images of 2.5 mm thickness were acquired using a LightSpeed RT16 CT scanner (GE Healthcare, Waukesha, WI). Images were sent to Eclipse version 8.9 (Varian Medical Systems, Palo Alto, CA) for delineation of the target volume and organs at risk (OARs), and dose calculation.

### Delineation of target volume and organs at risk

The clinical target volume (CTV) for the breast (CTV_Breast) was delineated in accordance with the European Society for Radiotherapy and Oncology guidelines [14], and included breast parenchyma that was distinguishable on simulation CT and could be wired via palpation. The planning target volume (PTV) for the breast (PTV_Breast) was defined as the CTV_Breast plus a 5-mm margin, and we trimmed the anterior border by 3 mm from the skin. The average PTV_Breast was 575.6 ± 231.4 cm^3^ (median: 520.7 cm^3^, range: 167.3–1110.2 cm^3^). The average equivalent sphere diameter was 10.1 ± 1.4 cm (median: 10.0 cm, range: 6.8–12.8 cm). The CTV for boost (CTV_Boost) was defined as operation scar plus a 15-mm margin, surgical clips plus a 10-mm margin, and seroma plus a 10-mm margin. The PTV for boost (PTV_Boost) was defined as the CTV_Boost plus a 5-mm margin, we trimmed the anterior border by 3 mm from the skin. The average PTV_Boost was 120.7 ± 46.9 cm^3^ (median: 120.2 cm^3^, range: 44.6–255.6 cm^3^). The average equivalent sphere diameter was 6.0 ± 0.8 cm (median: 6.1 cm, range: 4.4–7.9 cm). The average depth was 4.0 ± 0.9 cm (median: 4.0 cm, range: 2.5–6.7 cm). The average longest diameter was 8.3 ± 1.1 cm (median: 8.2 cm, range: 6.0–10.7 cm). The PTV1 was defined as the PTV_Breast minus the PTV_Boost, and the PTV2 was defined as the PTV_Boost. OARs such as a Heart, Lung, and spinal cord were also contoured.

### Prescription

The prescribed dose was 51 Gy in 30 fractions for the PTV1 and 60 Gy in 30 fractions for the PTV2. WBI planning that delivered 51 Gy to the whole breast (PTV1 + PTV2) was performed initially, and then 3 types of boost plans were made to deliver an additional 9 Gy to the tumor bed (PTV2).

### WBI planning

Two parallel-opposed tangential fields were used as the main fields, and to reduce hot spots and improve homogeneity, 2–4 subfields were usually added by using the field-in-field technique. We minimized the volume inside the PTV_Breast (PTV1 + PTV2) receiving >105% of the prescribed dose. For this plan, we used a 10 MV photon beam and a grid size of 2.5 mm. The analytic anisotropic algorithm (version 8.9.17) was used for the dose calculation. The superior and inferior borders of the field were set at the suprasternal notch and 2 cm below the inframammary fold, respectively. The medial border of the field was the midline of the patient, and the distance between the PTV and the border of multileaf collimator (MLC) on beam's eye view was set to be at least 5 mm.

### Boost planning

1. Electron 1 portal (E1P) plan: The margin for electron block was 5 mm from the PTV_Boost (PTV2). The electron energy was chosen depending upon the depth of the PTV_Boost (median: 16 MeV, range: 9–20 MeV). The source to skin distance for the electron beam was 100 cm. Electron Monte Carlo (version 8.9.08) with a grid size of 5 mm was used to calculate the electron dose. We intended to cover 100% of the PTV2 with > 90% of the prescribed dose and to minimize the volume inside the PTV_Boost receiving >105% of the dose. The final plan was developed to combine the boost plan and correspondent WBI plan.
2. Photon 3 portal (P3P) plan: The MLC margin was 5 mm from the PTV_Boost. This plan consisted of 3 fields in a hinge angle of 180° and the beam arrangement interval was 90° each. 6-MV and 10-MV photon energies were used. The wedge was not used for this plan. The grid size and the algorithm for the dose calculation were the same as those used in the WBI plan. Isocenter for this boost plan was set to be the same as that of the WBI plan to avoid shifting isocenters during treatment. The final plan was developed to combine the boost plan and correspondent WBI plan.
3. Photon 5 portal (P5P) plan: This plan consisted of 5 fields in a hinge angle of 200° and the beam arrangement interval was 50° each. The isocenter, photon energy, grid size, MLC margin, and algorithm for the dose calculation were the same as those used in the P3P plan. The final plan was developed to combine the boost plan and correspondent WBI plan.

### Parameters

For the PTV1 and PTV2, mean dose, V_40_ (V_n_: percentage of volume receiving more than at least *n* Gy), V_45_, V_50_, V_55_, and V_60_ were evaluated. For the total lung, mean dose, V_5_, V_10_, V_15_, V_20_, V_25_, and V_30_ were evaluated. For the heart, mean dose, V_10_, V_20_, V_30_, V_40_, and V_50_ were evaluated. For all ROIs, cumulated dose-volume histograms (DVHs) were compared. In the heart, DVH of left breast cases were additively compared, and for the total lung, DVH of right breast cases were also compared.

## Results

**Fig 1:**
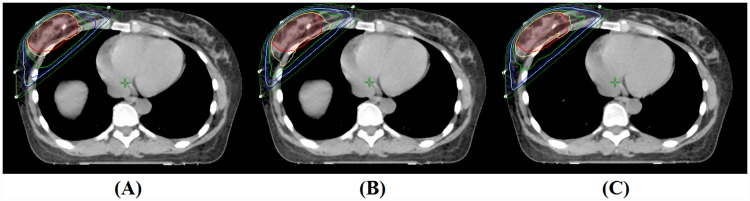
An example of dose distribution of (A) E1P plan, (B) P3P plan, and (C) P5P plan (PTV_Boost = semi-lucent red area; yellow line = 60 Gy; light green line = 57 Gy; blue line = 54 Gy; cyan line = 51 Gy; dark blue line = 48 Gy; green line = 30 Gy).

**Fig 2:**
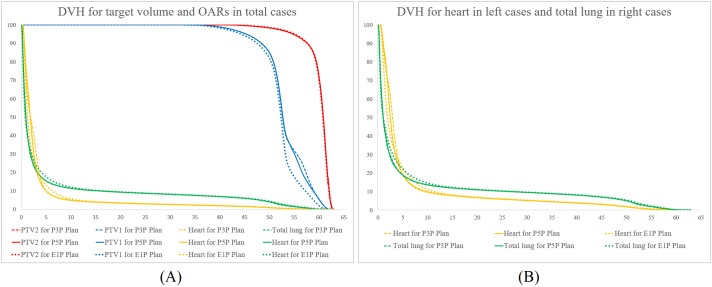
Comparison of average dose-volumetric histograms between the E1P, P3P, and P5P plans. (A) for the PTV1, PTV2, heart, and total lung in all cases, (B) for the heart in left breast cancer cases and total lung in right breast cancer cases.

Comparison of dosimetric parameters between 3 types of plans are summarized in Tables 1 and 2, and all values are presented in the manner of mean dose ± standard deviation. An example of dose distribution of (A) E1P plan, (B) P3P plan, and (C) P5P plan are shown in Fig 1, and average cumulative DVHs for the PTV1, PTV2, heart, and total lung are shown in Fig 2.

**Table 1:** Comparison of dosimetric parameters between the 3 types of plans in all 20 cases.

ROI	Parameters	E1P plan	P3P plan	P5P plan
PTV1	Mean dose (Gy)	60.0 ± 0.6	69.1 ± 0.6	60.1 ± 0.6
	V_40_ (%)	100.0 ± 0.1	100.0 ± 0.1	100.0 ± 0.1
	V_45_ (%)	99.7 ± 0.3	99.5 ± 0.5	99.6 ± 0.5
	V_50_ (%)	98.5 ± 1.5	98.2 ± 1.9	98.3 ± 1.8
	V_55_ (%)	95.0 ± 3.2	94.5 ± 3.4	94.7 ± 3.3
	V_60_ (%)	69.8 ± 8.8	71.5 ± 8.3	71.5 ± 9.3
PTV2	Mean dose (Gy)	52.1 ± 0.6	53.2 ± 0.8	53.1 ± 0.6
	V_40_ (%)	98.3 ± 0.7	98.8 ± 0.5	98.8 ± 0.6
	V_45_ (%)	94.1 ± 1.5	95.3 ± 1.3	95.3 ± 1.2
	V_50_ (%)	81.8 ± 3.8	84.7 ± 3.9	84.9 ± 3.3
	V_55_ (%)	17.5 ± 5.1	31.8 ± 10.2	30.1 ± 8.1
	V_60_ (%)	1.8 ± 0.9	5.4 ± 2.0	5.2 ± 1.6
Heart	Mean dose (Gy)	3.5 ± 2.7	3.6 ± 2.7	3.5 ± 2.7
	V_10_ (%)	5.6 ± 6.6	4.8 ± 5.9	4.9 ± 6.1
	V_20_ (%)	3.4 ± 4.4	3.3 ± 4.3	3.3 ± 4.3
	V_30_ (%)	2.6 ± 3.5	2.5 ± 3.5	2.6 ± 3.5
	V_40_ (%)	2.0 ± 2.8	1.9 ± 2.7	1.9 ± 2.7
	V_50_ (%)	1.0 ± 1.6	0.8 ± 1.5	0.9 ± 1.5
Total lung	Mean dose (Gy)	5.9 ± 1.7	5.7 ± 1.6	5.7 ± 1.6
	V_5_ (%)	17.6 ± 6.0	15.3 ± 4.9	15.5 ± 5.0
	V_10_ (%)	12.0 ± 3.8	11.3 ± 3.5	11.4 ± 3.5
	V_15_ (%)	10.3 ± 3.2	10.0 ± 3.1	10.1 ± 3.1
	V_20_ (%)	9.4 ± 3.0	9.2 ± 2.9	9.3 ± 2.8
	V_25_ (%)	8.8 ± 2.9	8.6 ± 2.8	8.6 ± 2.8
	V_30_ (%)	8.2 ± 2.8	8.1 ± 2.7	8.1 ± 2.7

Abbreviation: ROI = region of interest; E1P plan = electron 1 portal plan; P3P plan = photon 3 portal plan; P5P plan = photon 5 portal plan; V_n_ = percentage of volume receiving more than at least *n* Gy

**Table 2:** Comparison of dosimetric parameters between the 3 types of plans in terms of the heart and total lung.

ROI	Parameters	Left breast			Right breast		
		E1P plan	P3P plan	P5P plan	E1P plan	P3P plan	P5P plan
Heart	Mean dose (Gy)	5.6 ± 2.2	5.7 ± 2.2	5.6 ± 2.2	1.3 ± 0.3	1.4 ± 0.5	1.4 ± 0.4
	V_10_ (%)	11.0 ± 5.2	9.5 ± 4.9	9.8 ± 5.1	0	0	0
	V_20_ (%)	6.8 ± 3.6	6.6 ± 3.8	6.7 ± 3.9	0	0	0
	V_30_ (%)	5.3 ± 3.3	5.1 ± 3.3	5.1 ± 3.3	0	0	0
	V_40_ (%)	3.9 ± 2.7	3.7 ± 2.7	3.8 ± 2.7	0	0	0
	V_50_ (%)	1.9 ± 1.9	1.6 ± 1.8	1.7 ± 1.8	0	0	0
Total lung	Mean dose (Gy)	4.8 ± 1.0	4.7 ± 1.0	4.8 ± 1.1	7.0 ± 1.6	6.6 ± 1.5	6.7 ± 1.5
	V_5_ (%)	13.1 ± 2.7	11.8 ± 2.6	12.0 ± 2.7	22.1 ± 5.0	18.7 ± 4.3	18.9 ± 4.3
	V_10_ (%)	9.5 ± 2.2	9.1 ± 2.2	9.2 ± 2.2	14.5 ± 3.4	13.5 ± 3.1	13.6 ± 3.1
	V_15_ (%)	8.4 ± 2.0	8.2 ± 2.0	8.2 ± 2.0	12.2 ± 3.0	11.8 ± 2.9	11.9 ± 2.9
	V_20_ (%)	7.7 ± 2.0	7.6 ± 2.0	7.6 ± 2.0	11.9 ± 2.9	10.9 ± 2.8	10.9 ± 2.9
	V_25_ (%)	7.2 ± 1.9	7.1 ± 1.9	7.1 ± 1.9	10.3 ± 2.9	10.1 ± 2.8	10.3 ± 2.9
	V_30_ (%)	6.7 ± 1.8	6.6 ± 1.8	6.7 ± 1.8	9.6 ± 2.8	9.5 ± 2.8	9.5 ± 2.8

Abbreviation: ROI = region of interest; E1P plan = electron 1 portal plan; P3P plan = photon 3 portal plan; P5P plan = photon 5 portal plan; V_n_ = percentage of volume receiving more than at least *n* Gy

### Target volume coverage

In case of the PTV2, mean doses of the E1P, P3P, and P5P plans were 60.0 ± 0.6 Gy, 60.1 ± 0.6 Gy, and 60.1 ± 0.6 Gy, respectively, and the difference of mean dose between 3 plans was maximally 0.1 Gy. V_50_ of the E1P, P3P, and P5P plans were 98.5 ± 1.5%, 98.2 ± 1.9%, and 98.3 ± 1.8%, respectively, and V_55_ of the E1P, P3P, and P5P plans were 95.0 ± 3.2%, 94.5 ± 3.4%, and 94.7 ± 3.3%, respectively. The differences of V_50_ and V_55_ were maximally 0.3% and 0.5%, respectively.

In case of the PTV1, mean doses of the E1P, P3P, and P5P plan were 52.1 ± 0.6 Gy, 53.2 ± 0.8 Gy, and 53.1 ± 0.6 Gy, respectively, and the difference of mean dose between 3 plans was maximally 1.1 Gy. V_40_ of the E1P, P3P, and P5P plans were 98.3 ± 0.7%, 98.8 ± 0.5%, and 98.8 ± 0.6%, respectively, and V_45_ of the E1P, P3P, and P5P plans were 94.1 ± 1.5%, 95.3 ± 1.3%, and 95.3 ± 1.2%, respectively. The differences between 3 plans for V_40_ and V_45_, which are the parameters within prescription dose to the PTV1, were only 0.5% and 1.2%, respectively. V_55_ of the E1P, P3P, and P5P plans were 17.5 ± 5.1%, 31.8 ± 10.2%, and 30.1 ± 8.1%, respectively, and V_60_ of the E1P, P3P, and P5P plans were 1.8 ± 0.9%, 5.4 ± 2.0%, and 5.2 ± 1.6%, respectively. In case of V_55_ and V_60_, which are the parameters above the prescribed dose to the PTV1, the dose was lower in the E1P plan than that in the P3P and P5P plans, and the maximal differences were 14.3% and 3.6%, respectively. Because V_55_ and V_60_ of the PTV1 was affected by the plan for the PTV2, the lower values of V_55_ and V_60_ were adequate. Therefore, the E1P plan was considered more suitable than the P3P and P5P plans.

### Dose delivered to the heart and total lung

In case of the heart, comparisons between the 3 plans were performed using data from left breast cancer cases. Mean doses of the E1P, P3P, and P5P plans were 5.6 ± 2.2 Gy, 5.7 ± 2.2 Gy, and 5.6 ± 2.2 Gy, respectively, and the difference of mean dose between the 3 plans was maximally 0.1 Gy. In the range from V_10_ to V_50_ of the heart in the 3 plans, the value of each parameter was slightly higher in the E1P plan than in the P3P and P5P plans, and the difference between these values was less than 1.5%. The DVH curve also showed that the percentage of volume receiving less than 15 Gy was slightly higher in the E1P plan than that in the P3P and P5P plans, and the difference was small.

In case of the total lung, mean doses of the E1P, P3P, and P5P plans were 5.9 ± 1.7 Gy, 5.7 ± 1.6 Gy, and 5.7 ± 1.6 Gy, respectively. In the range from V_5_ to V_30_ of the total lung in the 3 plans, the value of each parameter was slightly higher in the E1P plan than in the P3P and P5P plans, and the difference between these values was less than 2%. The DVH curve showed that in the low dose area, the E1P plan was higher than the P3P and P5P plans, but the difference was minimal. When the right breast cancer cases were compared, the differences were higher. Mean doses of the E1P, P3P, and P5P plans were 7.0 ± 1.6 Gy, 6.6 ± 1.5 Gy, and 6.7 ± 1.5 Gy, respectively, and the difference was less than 0.4 Gy. In the range from V_5_ to V_30_ of the total lung in the 3 plans, the value of each parameter was also higher in the E1P Plan than in the P3P and P5P plans, and the difference was less than 3.5%. The DVH curve showed similar pattern.

## Discussion

The purpose of this study was to identify a more suitable boost plan for the SIB scheme in patients with breast cancer by comparing 3 types of WBI plans plus boost plans. According to the National Comprehensive Cancer Network (NCCN) guidelines, irradiation of 50 Gy in 25 fractions to the whole breast followed by delivering 8–16 Gy in 4–8 fractions to the tumor bed is currently recommended. Although other options such as partial breast irradiation or hypofractionated RT exist, our comparative study was designed to develop treatment plans for WBI plus a boost to the tumor bed by using a modified dose schedule based on the conventional fractionation scheme. In the SIB technique, the fraction number of irradiation to the whole breast and tumor bed needs to be same. Therefore, the fraction size should be modified according to the biologically effective dose (BED) of the conventional fractionation scheme. The prescribed dose to the PTV1 was set to be 51 Gy in 30 fractions, and the BED was calculated as 59.7 Gy_10_, which is similar to 60 Gy_10_ of 50 Gy in 25 fractions. In case of the PTV2, the prescribed was 60 Gy in 30 fractions, which is the same as the recommended scheme per the NCCN guidelines. Therefore, no modifications were necessary.

In previous studies regarding the boost planning, various boost plans were compared without being combined with the WBI plan [9–13]. These studies concluded that the multi-field 3D-CRT, IMRT, or VMAT were superior to the electron therapy with respect to the target volume coverage and normal tissue sparing. Tosca et al. reported that the electron therapy showed the lowest coverage and the largest interpatient variability with respect to the target coverage and dose inhomogeneity when compared with the 3D-CRT, DCAT, IMRT, and proton therapy [12]. According to Van Parijs et al., the electron therapy showed the worst PTV coverage and delivered a higher dose to the ipsilateral lung and heart when compared with other techniques [13]. However, these conclusions could change if the boost plans are evaluated together with the WBI plan.

In this study, we concluded that a boost plan using electrons was more suitable than 2 other plans using multi-field photon energy. Our results showed that the doses delivered to the heart and lung were slightly higher. However, in case of the heart, the difference in V_10_ was less than 1% and there was no difference in the mean dose. From V_20_ to V_50_, the difference was less than 0.1%. In case of the heart dose in patients with left breast cancer, the difference in V_10_ and mean dose was maximally 1.5% and 0.1%, respectively. In case of the total lung, the difference in V_5_ and V_10_ was 2% and less than 1%, respectively, and the difference in the mean dose was only 0.2%. In case of the lung dose in patients with right breast cancer, the difference in V_5_ and V_10_ was 3.4% and 1%, respectively. According to the Quantitative Analyses of Normal Tissue Effects in the Clinic (QUANTEC) guidelines regarding the heart, a V_25_ <10% (in 2 Gy per fraction) is associated with a <1% probability of cardiac mortality ~15 years after RT [15]. Although our data showed that V_20_ of the heart in the electron plan was 0.1–0.2% higher than that in the photon plans, V_20_ was 6.8%, which is lower and safer than the 10% value recommended by the QUANTEC guidelines. In case of the total lung, the guidelines suggests that it is prudent to limit V_20_ to <30–35% and mean lung dose to <20–23 Gy with conventional fractionation [16]. Although our data showed that V_25_ and mean dose of the total lung in the electron plan was 0.2% higher than those in the photon plans, 8.8% of V_25_ and 5.9 Gy of mean dose are within the safety range when compared with the recommended dose constraints in the QUANTEC guidelines. Therefore, it is inappropriate that the electron therapy is deemed unsuitable for boosts owing to minimal inferiority. If the dose delivered to the heart and lung is not acceptable for treatment, it is mainly owing to the WBI plan, not because of the boost plan. Thus, the WBI plan should be modified in such circumstances because the required dose to boost was approximately 15–25% of the dose that was needed for adjuvant RT.

For the PTV2 (PTV_Boost), all the 3 plans showed similar target volume coverage. In case of the PTV1 (PTV_Breast minus PTV_Boost), we preferred the electron plan to the other photon plans. In the dose range over the prescribed dose (51 Gy), V_55_ and V_60_ were much higher in the photon plans that in the electron plan. This is due to an unnecessary increase of the dose in the normal breast parenchyma, which resulted from multiple beams of the photon plans. In addition, in case of a medially located tumor bed, the electron plan was preferred because a portion of contralateral breast was included in the photon plans. However, because the electron tray should be repeatedly attached and detached in every treatment session, the efficacy could be reduced in terms of treatment time. In the deeply located tumor bed, well optimized photon or proton therapy was preferred owing to the limitation of penetration of electrons [12].

In the SIB technique, displacement of the tumor bed and changing the lumpectomy cavity should be considered. Won et al. performed a comparison between simulation CT and fraction ultrasound imaging to evaluate the displacement of the tumor bed [17]. The overlap of the tumor bed was 78% and the mean absolute radial displacement was 10.8 mm. Chen et al. evaluated the change in lumpectomy cavity by comparing findings from simulation CT and fraction CT, which were performed in every treatment session [18]. The relative volume ratio was 29–138%, and maximum overlap ratio was 29–86%. For most patients, the lumpectomy cavity volumes decreased, except for the 3 cases in which increases were observed. The change includes an initial volume increase due to seroma filling, followed by a decrease due to seroma absorption. Therefore, a sufficient PTV margin for boost was needed. However, in case of the repetitive aspiration of the seroma and large volume of the seroma, the size of seroma could change considerably during the treatment period. In such cases, the SIB may not be appropriate because of the possibility of missing the tumor bed or inclusion of unnecessary breast tissues in the treatment field.

In conclusion, a simple electron plan for the tumor bed boost is more suitable than multi-field photon plans in terms of the target volume coverage and normal tissue sparing. However, because the photon plans also showed clinically acceptable quality for boost, photon plans may be suitable for individual cases. Furthermore, when using the SIB technique, cautious patient selection and determination of proper margins for PTV are needed to avoid missing the tumor bed.

## References

[1] Ghoncheh M, Pournamdar Z, Salehiniya H. Incidence and Mortality and Epidemiology of Breast Cancer in the World. Asian Pacific journal of cancer prevention: APJCP. 2016;17 Spec No:43–6. 10.7314/apjcp.2016.17.s3.43 27165206

[2] Clarke M, Collins R, Darby S, Davies C, Elphinstone P, Evans V, et al Effects of radiotherapy and of differences in the extent of surgery for early breast cancer on local recurrence and 15-year survival: an overview of the randomised trials. Lancet. 2005;366(9503):2087–106. 10.1016/S0140-6736(05)67887-7 16360786

[3] Fisher B, Anderson S, Bryant J, Margolese RG, Deutsch M, Fisher ER, et al Twenty-year follow-up of a randomized trial comparing total mastectomy, lumpectomy, and lumpectomy plus irradiation for the treatment of invasive breast cancer. N Engl J Med. 2002;347(16):1233–41. 10.1056/NEJMoa022152 12393820

[4] Sautter-Bihl ML, Sauer R. Once more confirmed: adjuvant radiotherapy and improved local control provide a significant survival benefit for early breast cancer patients. Strahlenther Onkol. 2006;182(4):199–201. 10.1007/s00066-006-6701-4 16622620

[5] Bartelink H, Horiot JC, Poortmans PM, Struikmans H, Van den Bogaert W, Fourquet A, et al Impact of a higher radiation dose on local control and survival in breast-conserving therapy of early breast cancer: 10-year results of the randomized boost versus no boost EORTC 22881–10882 trial. J Clin Oncol. 2007;25(22):3259–65. 10.1200/JCO.2007.11.4991 17577015

[6] Aly MM, Glatting G, Jahnke L, Wenz F, Abo-Madyan Y. Comparison of breast simultaneous integrated boost (SIB) radiotherapy techniques. Radiat Oncol. 2015;10:139 10.1186/s13014-015-0452-2 26156086 PMC4495684

[7] Guerrero M, Li XA, Earl MA, Sarfaraz M, Kiggundu E. Simultaneous integrated boost for breast cancer using IMRT: a radiobiological and treatment planning study. Int J Radiat Oncol Biol Phys. 2004;59(5):1513–22. 10.1016/j.ijrobp.2004.04.007 15275739

[8] Wu S, Lai Y, He Z, Zhou Y, Chen S, Dai M, et al Dosimetric comparison of the simultaneous integrated boost in whole-breast irradiation after breast-conserving surgery: IMRT, IMRT plus an electron boost and VMAT. PLoS One. 2015;10(3):e0120811 10.1371/journal.pone.0120811 25781183 PMC4363530

[9] Chen GP, Liu F, White J, Vicini FA, Freedman GM, Arthur DW, et al A planning comparison of 7 irradiation options allowed in RTOG 1005 for early-stage breast cancer. Med Dosim. 2015;40(1):21–5. 10.1016/j.meddos.2014.06.007 25155215 PMC4654615

[10] Park K, Lee Y, Cha J, You SH, Kim S, Lee JY. Influence of different boost techniques on radiation dose to the left anterior descending coronary artery. Radiation oncology journal. 2015;33(3):242–9. 10.3857/roj.2015.33.3.242 26484308 PMC4607578

[11] Park SH, Kim JC. Comparison of electron and x-ray beams for tumor bed boost irradiation in breast-conserving treatment. J Breast Cancer. 2013;16(3):300–7. 10.4048/jbc.2013.16.3.300 24155759 PMC3800726

[12] Toscas JI, Linero D, Rubio I, Hidalgo A, Arnalte R, Escude L, et al Boosting the tumor bed from deep-seated tumors in early-stage breast cancer: a planning study between electron, photon, and proton beams. Radiother Oncol. 2010;96(2):192–8. 10.1016/j.radonc.2010.05.007 20538361

[13] Van Parijs H, Reynders T, Heuninckx K, Verellen D, Storme G, De Ridder M. Breast conserving treatment for breast cancer: dosimetric comparison of different non-invasive techniques for additional boost delivery. Radiat Oncol. 2014;9:36 10.1186/1748-717X-9-36 24467916 PMC3907792

[14] Offersen BV, Boersma LJ, Kirkove C, Hol S, Aznar MC, Sola AB, et al ESTRO consensus guideline on target volume delineation for elective radiation therapy of early stage breast cancer, version 1.1. Radiother Oncol. 2016;118(1):205–8. 10.1016/j.radonc.2015.12.027 26791404

[15] Gagliardi G, Constine LS, Moiseenko V, Correa C, Pierce LJ, Allen AM, et al Radiation dose-volume effects in the heart. Int J Radiat Oncol Biol Phys. 2010;76(3 Suppl):S77–85. 10.1016/j.ijrobp.2009.04.093 20171522

[16] Marks LB, Bentzen SM, Deasy JO, Kong FM, Bradley JD, Vogelius IS, et al Radiation dose-volume effects in the lung. Int J Radiat Oncol Biol Phys. 2010;76(3 Suppl):S70–6. 10.1016/j.ijrobp.2009.06.091 20171521 PMC3576042

[17] Wong P, Muanza T, Reynard E, Robert K, Barker J, Sultanem K. Use of three-dimensional ultrasound in the detection of breast tumor bed displacement during radiotherapy. Int J Radiat Oncol Biol Phys. 2011;79(1):39–45. 10.1016/j.ijrobp.2009.10.023 20621425

[18] Chen X, Qiao Q, DeVries A, Li W, Currey A, Kelly T, et al Adaptive replanning to account for lumpectomy cavity change in sequential boost after whole-breast irradiation. Int J Radiat Oncol Biol Phys. 2014;90(5):1208–15. 10.1016/j.ijrobp.2014.08.342 25442046

